# Prebiotic synthesis of mineral-bearing microdroplet from inorganic carbon photoreduction at air–water interface

**DOI:** 10.1093/pnasnexus/pgad389

**Published:** 2023-11-15

**Authors:** Qiuyue Ge, Yangyang Liu, Wenbo You, Wei Wang, Kejian Li, Xuejun Ruan, Lifang Xie, Tao Wang, Liwu Zhang

**Affiliations:** Shanghai Key Laboratory of Atmospheric Particle Pollution and Prevention, National Observations and Research Station for Wetland Ecosystems of the Yangtze Estuary, IRDR International Center of Excellence on Risk Interconnectivity and Governance on Weather, Department of Environmental Science and Engineering, Fudan University, Shanghai 200433, P. R. China; Shanghai Key Laboratory of Atmospheric Particle Pollution and Prevention, National Observations and Research Station for Wetland Ecosystems of the Yangtze Estuary, IRDR International Center of Excellence on Risk Interconnectivity and Governance on Weather, Department of Environmental Science and Engineering, Fudan University, Shanghai 200433, P. R. China; Shanghai Key Laboratory of Atmospheric Particle Pollution and Prevention, National Observations and Research Station for Wetland Ecosystems of the Yangtze Estuary, IRDR International Center of Excellence on Risk Interconnectivity and Governance on Weather, Department of Environmental Science and Engineering, Fudan University, Shanghai 200433, P. R. China; Shanghai Key Laboratory of Atmospheric Particle Pollution and Prevention, National Observations and Research Station for Wetland Ecosystems of the Yangtze Estuary, IRDR International Center of Excellence on Risk Interconnectivity and Governance on Weather, Department of Environmental Science and Engineering, Fudan University, Shanghai 200433, P. R. China; Shanghai Key Laboratory of Atmospheric Particle Pollution and Prevention, National Observations and Research Station for Wetland Ecosystems of the Yangtze Estuary, IRDR International Center of Excellence on Risk Interconnectivity and Governance on Weather, Department of Environmental Science and Engineering, Fudan University, Shanghai 200433, P. R. China; Shanghai Key Laboratory of Atmospheric Particle Pollution and Prevention, National Observations and Research Station for Wetland Ecosystems of the Yangtze Estuary, IRDR International Center of Excellence on Risk Interconnectivity and Governance on Weather, Department of Environmental Science and Engineering, Fudan University, Shanghai 200433, P. R. China; Shanghai Key Laboratory of Atmospheric Particle Pollution and Prevention, National Observations and Research Station for Wetland Ecosystems of the Yangtze Estuary, IRDR International Center of Excellence on Risk Interconnectivity and Governance on Weather, Department of Environmental Science and Engineering, Fudan University, Shanghai 200433, P. R. China; Shanghai Key Laboratory of Atmospheric Particle Pollution and Prevention, National Observations and Research Station for Wetland Ecosystems of the Yangtze Estuary, IRDR International Center of Excellence on Risk Interconnectivity and Governance on Weather, Department of Environmental Science and Engineering, Fudan University, Shanghai 200433, P. R. China; Shanghai Key Laboratory of Atmospheric Particle Pollution and Prevention, National Observations and Research Station for Wetland Ecosystems of the Yangtze Estuary, IRDR International Center of Excellence on Risk Interconnectivity and Governance on Weather, Department of Environmental Science and Engineering, Fudan University, Shanghai 200433, P. R. China; Shanghai Institute of Pollution Control and Ecological Security, Shanghai 200092, P. R. China

**Keywords:** air–water interface, microdroplet, origin of life, sphalerite minerals, probiotic chemistry

## Abstract

The origin of life on Earth is an enigmatic and intricate conundrum that has yet to be comprehensively resolved despite recent significant developments within the discipline of archaeology and geology. Chemically, metal-sulfide minerals are speculated to serve as an important medium for giving birth in early life, while yet so far direct evidence to support the hypothesis for the highly efficient conversion of inorganic carbon into praxiological biomolecules remains scarce. In this work, we provide an initial indication that sphalerite, employed as a typical mineral, shows its enormous capability for promoting the conversion of inorganic carbon into elementary biomolecule formic acid (HCOOH) in airborne mineral-bearing aerosol microdroplet, which is over two orders of magnitude higher than that of the corresponding conventional bulk-like aqueous phase medium in the environment (e.g. river, lake, sea, etc.). This significant enhancement was further validated by a wide range of minerals and clays, including CuS, NiS, CoS, CdS, MnS, elemental sulfur, Arizona Test Dust, loess, nontronite, and montmorillonite. We reveal that the abundant interface of unique physical–chemical features instinct for aerosol or cloud microdroplets reduces the reaction energy barrier for the reaction, thus leading to extremely high HCOOH production (2.52 × 10^14^ kg year^−1^). This study unfolds unrecognized remarkable contributions of the considered scheme in the accumulation of prebiotic biomolecules in the ancient period of the Earth.

**Significance Statement**

The origin of life remains an enigmatic puzzle. Conversion of inorganic carbon to small HCOOH organic molecules is crucial as they are the basic building blocks for intricate compounds and primitive life. Here, we observed the extremely fast conversion process of inorganic carbon to organic one initiated by airborne microdroplets suspended with sphalerite mineral. More critically, the process can be a great contributor to the origin of life, leading to a previously unrecognized considerable production rate of HCOOH. This natural solar-driven inorganic carbon conversion emphasizes the important role of air–water interface coupling of minerals in fostering prebiotic biomolecules in the primitive Earth’s environment.

## Introduction

The origin of life remains an elusive and complex issue that has yet to be fully revealed. It is generally accepted that the conversion of inorganic carbon into simple organic molecules (formic acid, acetic acid, pyruvate, and amino acids) is a pivotal step, as it provides the fundamental building blocks for more complex biological compounds, which ultimately develop into primitive life forms (1–5). Among them, formic acid (HCOOH) receives great attention due to the priority of reduced carbon intermediate in route to methyl synthesis (6). However, the production of this basic organic molecule HCOOH through a conventional slow heterogeneous catalytic process fails to explain the flourishing life origin by the time-consuming evolution of pieces of biomolecule blocks (days or weeks) within their short life span (several hours) when exposed to the rugged environment (4, 7, 8). Therefore, a fast formation pathway that goes beyond the known conventional reaction channel does exist, which is a critical step to unfold the unresolved life-origin mystery disputed for decades.

Outstanding chemical reactivity at air–water interfaces is alluring and receives tremendous interest across diverse disciplines (9–25). The hydrogen bond network at the air–water interface is inevitably disrupted, and the contrasting chemical and physical properties of molecules present at the interface (such as hydrogen bonds, dipole moments, and acidity) extremely differ from those in the bulk, which can result in significantly faster reaction kinetics at the air–water interface when compared with reactions occurring in bulk aqueous media. Recently, universal airborne microdroplet particles (e.g. aerosol particles, cloud water, etc.), which possess abundant air–water interfaces, have attracted tremendous attention for their great impact on altering the reaction efficiencies in a wide range of atmospheric relevant reactions (8, 16, 25–31). The atmospheric aerosol microdroplets have been proposed as prebiotic chemical reactors (8, 31), and some spontaneous reactions relevant to the probiotic chemistry in aqueous microdroplets’ gas–liquid interface have been documented. For instance, phosphorylation proceeds spontaneously within aqueous microdroplets containing a mixture of sugars and phosphoric acid (32), and the “automatic” synthesis of uridine and other ribonucleotides (33), as well as the formation of peptide bonds during the condensation process of leucine ethyl ester are available in the presence of Cu^2+^ ions at the gas–liquid interface (34). The above information highlights the nonmarginal role of a natural airborne “microdroplet” reaction chamber in prebiotic chemistry.

On the other hand, the catalog of the minerals in the earliest Hadean period was believed to mainly comprise 80 S-rich minerals (35), and recent archaeology studies also observed these oldest mineral deposits such as the mid-Paleozoic Yukon-Tanana terrane, the northern Canadian Cordillera (36), Chelopech Ore Field (Bulgaria) (37), and Palaeoproterozoic Kerry Road (38), suggesting that semiconducting sulfur-bearing minerals are ubiquitous in primitive Earth’s environment (39). Alternative geology evidence in support of the origin of life that is linked to the crustal mineral lies in the low-eruptive volcanic caldera on continental land in Western Australia (about 3.48 billion years ago) where lasting voluminous hydrothermal fluid circulation activities were at play (40) and have been postulated as pivotal agents in the prebiotic processes across various natural circumstances. Excitingly, these minerals have been demonstrated to show outstanding capability in inorganic carbon photoreduction, thus leaving them considered as fundamental components in the prebiotic processes, often referred to as “proto-enzymes” orchestrating prebiotic organic syntheses. They have also played a pivotal role in sustaining the continuous chemical evolution, contributing to the functionality of intricate biochemical systems (41–45). In the primitive Earth’s environment, minerals can be brought to the ground by volcanic eruptions, and some of them are carried into the air by wind currents (7, 46–48). Notably, the CO_2_ concentration level of the primordial atmosphere was perhaps 100–1,000 times higher than that in the current one, and early aqueous environments can consequently accumulate a considerable amount of (bi)carbonates anions, dominated in the form of HCO_3_^−^ through dissolution equilibrium of atmospheric CO_2_ (49) in the acidic environment. Additionally, in the past tens to hundreds of millions of years, there have been infinitely many rounds of cycles (referring to the continuous production and sink processes of the airborne aerosol microdroplets that are derived from the aquatic environment enriched with bicarbonate ions through CO_2_ respiratory, e.g. ocean), with their average lifetime ranging from days to months (8). From this perspective, the emitted mineral dust particles that come from a series of geological activities (50–52) (e.g. volcanism, earthquakes, landslides, mineral weathering, etc.) can enter into atmospheric microdroplets, forming a natural microdroplet reactor full of these photoreactive mineral particles, where photocatalytic reduction of inorganic carbon to these organic building block molecules mediated by such “natural photocatalyst” takes place (53).

Motivated by the above important findings and information, the synergistic interplay between the excellent solar-driven organification trigged by metallic sulfide minerals and unique interfacial properties of airborne microdroplets is speculated to take place, and a large quantity of HCOOH is thus expected to form in the natural aerosol full of suspended dust particles, which is far beyond preceding considered reaction medium that either belongs to the solid heterogeneous surface or pristine air–water interface. This special microenvironment potentially serves as an important pathway for the transformation of inorganic carbon to organic carbon and consequently contributes to key organic moieties for the origin of life. Understanding such pathways and complicated environments that can facilitate the emergence of these primitive HCOOH organic molecules holds significant importance (42, 43, 54, 55). Yet, to our best knowledge, rare information is currently available for the prebiotic synthesis reactions catalyzed by the airborne minerals in the atmospheric microdroplet system, and to what extent their reactivity would be altered has not been reported.

In this study, we employed sphalerite as a typical sulfur-bearing mineral to investigate its capacity for photoreduction of inorganic carbon (referring to the gas-phase CO_2_ and aqueous-derived bicarbonate anion) to organic small molecules within airborne microdroplets and further evaluate the significant contribution of photochemical reduction of inorganic carbon by mineral-embedded microdroplet to the origin of life. The possible mechanism behind the extremely high HCOOH yield (approximately two orders of magnitude relative to the bulk) has been unfolded, and the photocatalytic reduction of inorganic carbon in microdroplets by other geologically rich metal sulfides has been investigated as well. This fast solar-driven natural inorganic carbon reduction reaction pathway in an airborne “microdroplet” reaction chamber suspended with minerals reveals the previously unrecognized route of significance that creates considerable prebiotic biomolecules for lives in the early Earth environment.

## Results

### Characterization of minerals

**Fig. 1.**
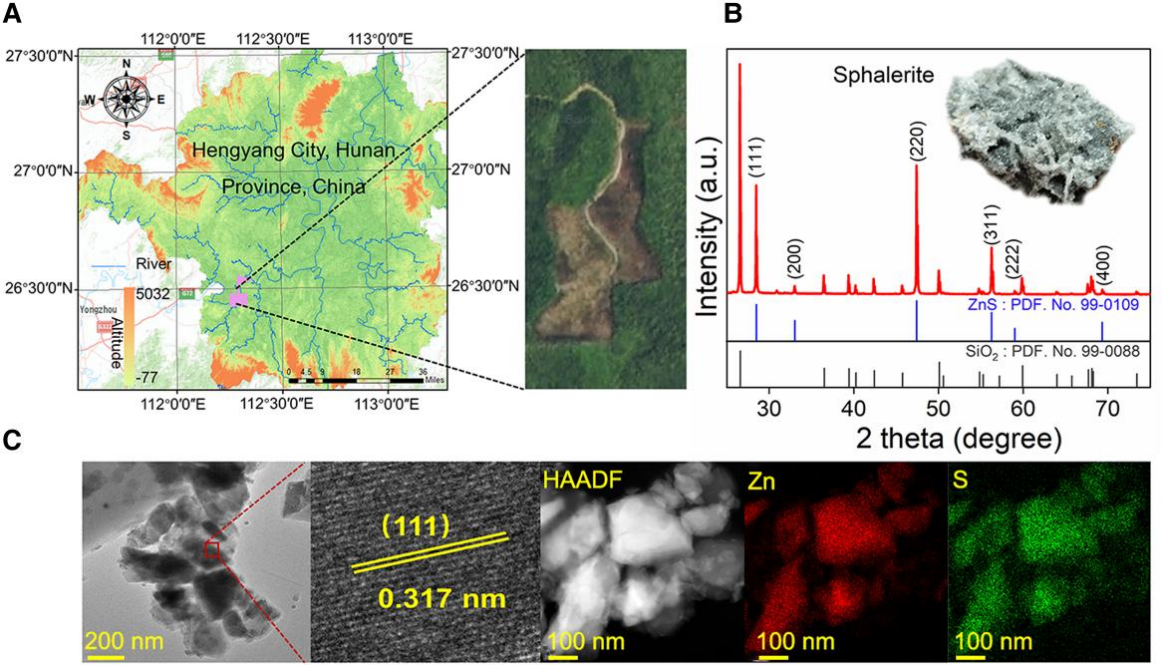
Characterization of sphalerite. A) Geographic position of the natural sphalerite source. B) XRD pattern. C) Corresponding bright-field TEM, HAADF images, and EDS analysis for natural ZnS minerals.

The natural sphalerite samples were derived from Hengyang City in Hunan Province, China, with a specific geographic position depicted in Fig. 1A. Prior to characterization and subsequent photoactivity measurement, the samples were pretreated using ball milling to mimic the natural polishing process of dust particles due to their geological activity. Subsequently, we carefully applied ultrasonic cleaning approach to remove undesired organic impurities to avoid complicated high-level backgrounds over the minerals (56). Powder X-ray diffraction (Fig. 1B) results indicate that the main components of the mineral were sphalerite-phase ZnS (PDF no. 5-0566) and quartz-phase SiO_2_ (PDF no. 46-1045). The mineral sample exhibited lattice fringes of 0.317 nm, corresponding to the (111) crystal plane of sphalerite-phase ZnS (57), as illustrated by bright-field transmission electron microscopy (TEM) and high-angle annular dark field (HAADF) images (Fig. 1C). Elemental mapping analysis using energy-dispersive X-ray spectroscopy (EDS) demonstrated that Zn and S were homogeneously distributed throughout the mineral sample (Fig. 1C), thus enabling a good photocatalytic performance observed in artificial photocatalysts, as well evidenced by scanning electron microscopy analysis (Fig. S1). To further confirm the contribution of ZnS in the generation of basic organic molecules necessary for prebiotic biomolecules, we synthesized pure artificial ZnS using an early developed method (58) for good reference and comparison. The crystal phase of the synthesized ZnS is characterized as sphalerite-phase ZnS, with the main exposed crystal facet being (111), as indicated by XRD patterns and TEM images (Fig. S2).

### The photoactivity test of ZnS

**Fig. 2.**
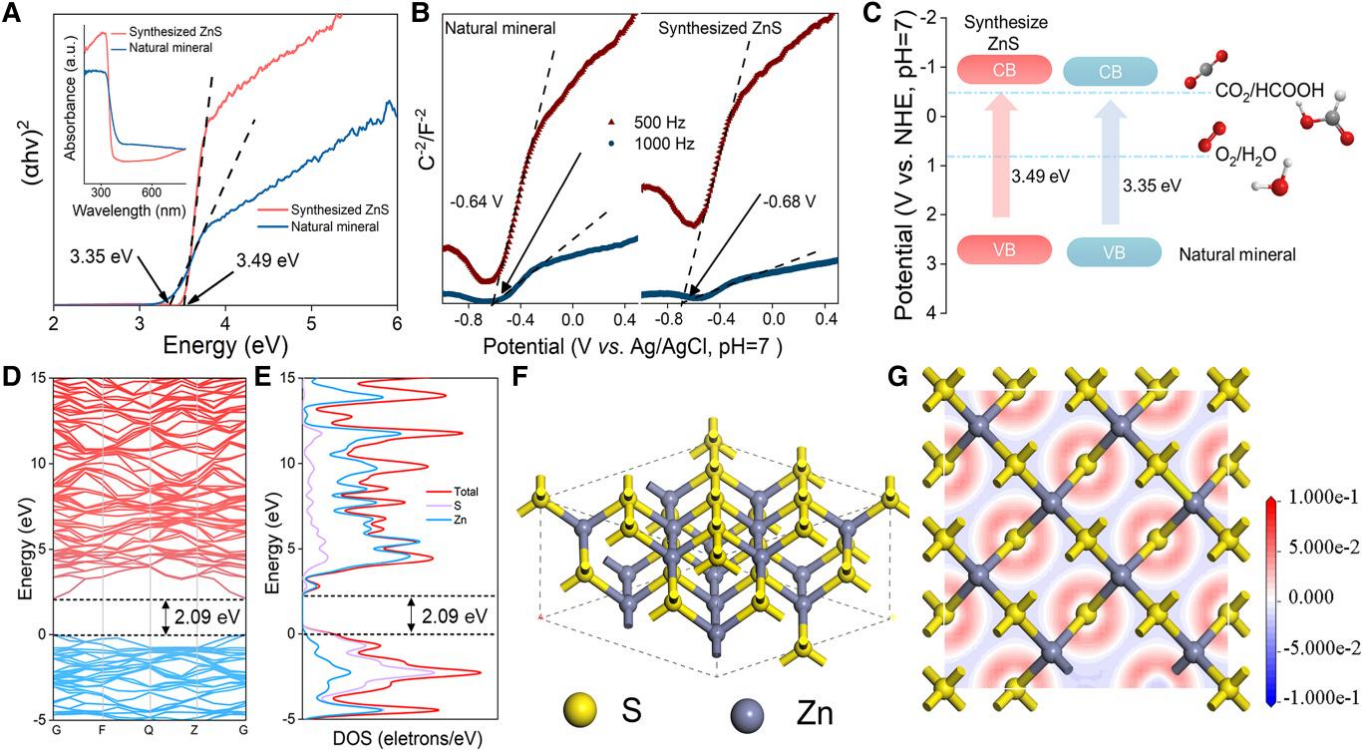
Semiconducting properties and optical spectroscopy of ZnS. A) The obtained bandgaps of ZnS (inset: UV–Vis DRS). B) Mott−Schottky plots. C) Schematic illustration of the electronic band structures. D) Band structure. E) The DOSs. F) Overview of crystal structure. G) Electron density difference.

Solid-state UV–vis diffuse reflectance spectra (UV–Vis DRS) analysis reveals that the natural ZnS mineral is 3.35 eV (Fig. 2A), slightly narrower than that of the synthesized ZnS (3.49 eV), with the details of the analysis and experiments shown in the Materials and methods section. Mott–Schottky measurement (Fig. 2B) confirms that natural and synthesized ZnS are featured in N-type semiconducting, with their flat-band potentials at −0.64 and −0.68 V (vs. Ag/AgCl; pH = 7), and conduct band minimums (CBMs) are accordingly determined to be at −0.64 and −0.68 eV, respectively, considering that the flat-band potential of N-type semiconducting is generally about 0.2 V more positive than its CBM (59, 60). We systematically investigated the band structure and density of states (DOSs) for ZnS by density-functional theory (DFT) calculation using local-density approximation (LDA) as exchange correlational functionals (61). Clearly, Fig. 2D and E reveals that ZnS is an N-type semiconducting with a direct band gap of 2.09 eV, in good agreement with the literature (62), but narrower than the experimental results probably because the calculated value within the density-functional LDA might be underestimated (63). The Perdew, Burke, and Ernzerhof, generalized gradient approximation total and partial electronic DOS indicated the density states at the CBM are mainly contributed by the S 3p-orbital, whereas those in the valence band maximum are mainly by the 3d-orbital of Zn (Fig. 2F and G). Overall, ZnS shows appropriate band edge positions in their electronic structures, which is essentially favorable for the catalytic inorganic carbon reduction (Fig. 2C). These electronic properties lead us to speculate ZnS as a natural semiconducting mineral that is at play in the origin of life (2).

### The fast solar-driven transformation of inorganic carbon to HCOOH in microdroplets that go beyond the bulk

**Fig. 3.**
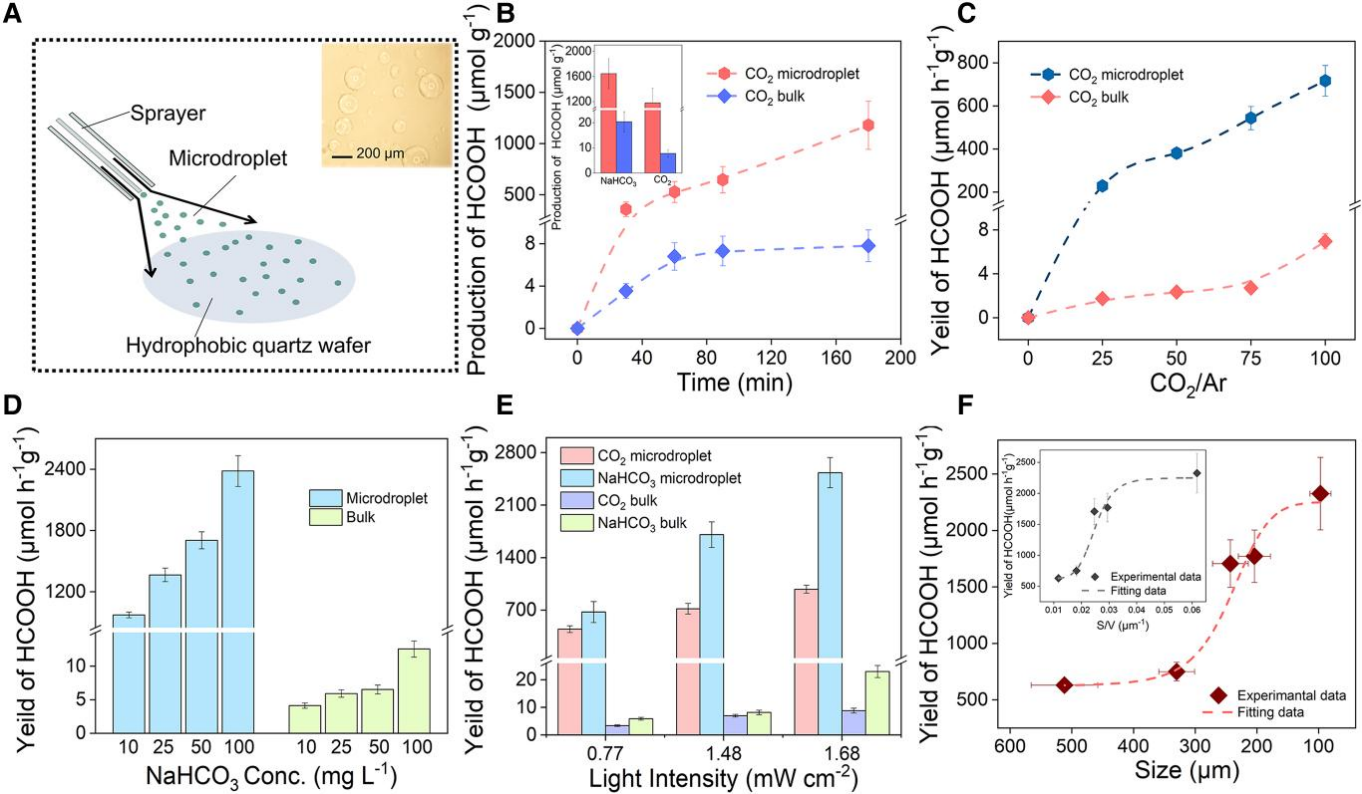
The HCOOH production varied with the following conditions in the microdroplet reaction system. A) Schematic diagram of the microdroplets produced (inset: microscopic image of the produced microdroplets). B) Photoreduction inorganic carbon reaction under UV-lamp radiation (inset: the yield of HCOOH under different carbon sources after 180 min). The influences of C) CO_2_ concentration (0, 25, 50, 100%), D) NaHCO_3_ concentration on photoreduction reaction (10, 25, 50, and 100 mM; UV-lamp radiation; room temperature; 0.8 g L^−1^ ZnS), E) light irradiation intensity, and F) microdroplet size (inset: the correlation between the yield of HCOOH and the microdroplet *S*/*V*).

Schematically, the microdroplet was produced utilizing a sprayer (Fig. 3A) on the superhydrophobic quartz glass substrate and then transferred into a homemade reaction chamber. Gas of high relative humidity (∼90%) was introduced into the chamber for 10 min to stabilize the microdroplet before reaction, and ZnS was uniformly dispersed in the microdroplets even after 180 min, with the negligible change of the size, thus verifying the feasibility of solar-driven reaction in microdroplets (Figs. S3 and S4).

The HCOOH gradually builds up as the photoreduction reaction proceeds (Figs. 3B and S8). After 180 min, the HCOOH production in the microdroplet system reaches up to ca. 1,304 μmol g^−1^ (gaseous CO_2_) and 2,634 μmol g^−1^ (25 mM NaHCO_3_ solution), which is over two orders of magnitude higher than that of 7 μmol g^−1^ (gaseous CO_2_) and 20 μmol g^−1^ (25 mM NaHCO_3_ solution) in the corresponding bulk phase reaction. Control experiments reveal that in the absence of ZnS, UV-light, or C-source, the concentration of the products except HCOOH is negligible (Fig. S8), which is below the detection limit of gas chromatography (GC) and ion chromatography (IC) analyses, indicating that HCOOH is the main product that comes from the photoreduction reaction in the natural environment. More importantly, the cooperation mechanism between ZnS dust and air–water interface for HCOOH production (>700 μmol h^−1^ g^−1^, Fig. S5) can be deduced for ZnS-bearing microdroplet system, which is extremely larger than HCOOH yields observed for the pristine ZnS mineral (∼10 μmol h^−1^ g^−1^) water microdroplet (not detected) and bulk ZnS suspension systems (∼10 μmol h^−1^ g^−1^). Additionally, in the microdroplet system, the HCOOH yield increases from 228 to 717 μmol h^−1^ g^−1^ when the CO_2_/Ar ratio increases from 25 to 100% (Fig. 3C). The production can be further increased to 2,382 μmol h^−1^ g^−1^ by increasing the initial concentration of inorganic carbon source, e.g. in the presence of 100 mM HCO_3_^−^ (Fig. 3D). In the bulk phase reaction, the HCOOH yield was nearly two orders of magnitude lower than the microdroplet reaction under different reaction conditions, indicating that the microdroplet reaction system has superior photochemical reaction performance. These inorganic carbon conversion reactions are positively correlated with light intensity (64, 65), whereas the HCOOH yield in the microdroplet reaction system significantly exceeds the bulk phase system even in the low-light intensity experiment (0.77 mW cm^−2^, Fig. 3E). The incident photon-to-current conversion efficiency (IPCE) in the microdroplet system under 310 nm irradiation was calculated to be 3.04% (1.48 mW cm^−2^). To confirm the carbon source, we further conducted isotope light experiments using ^13^CO_2_ (3 h) through in situ Raman analysis (Fig. S9), the C=O stretching of ^12^COOH at 1,648 cm^−1^ was shifted to 1,635 cm^−1^, consistent with the Raman shift trend in early observation (66). This result indicates that HCOOH comes from the introduction of an inorganic carbon source rather than the exogenous carbon contamination in this reaction system. As the surface area-to-volume ratio (*S*/*V*) increases, namely the reduction of the size of microdroplets, the ratio of interface region to interior region in microdroplets will be further enlarged (67). Clearly, HCOOH yield is positively correlated to the size of the microdroplets (Fig. 3F), suggesting that this crucial chemical process associated with the origin of life directly links to the abundant interface of the microdroplets. Size-dependent HCOOH yield kinetics was further validated in individual microdroplets of known size using the Raman analysis, where a confocal Raman spectroscopy coupled with an optical microscope (RENISHAWS inVia Raman microscope) was applied to determine HCOOH yield using the established calibration curve. Specifically, HCOOH has characteristic features of vibrational bands for *V_s_*(C–H) at ∼2,950 cm^−1^, *V*(H_2_O) at ∼3,420 cm^−1^, and the intensity ratio *V_s_*(C–H)/*V*(H_2_O) exhibits a good linear relationship with HCOOH concentration (Fig. S10A and B). On this basis, we quantified the HCOOH yield in the microdroplet reaction system and our quantification results show a significant increase in HCOOH yield as the microdroplet diameter decreases from 534 to 10 μm, with an enhancement factor approximating 32 times. This agrees with the trend observed for the yield of HCOOH in microdroplets ranging from 100 to 500 μm by employing the IC method (Fig. S10C). All these observations provide valuable insights into the relationship between microdroplet size and HCOOH yield. This is consistent with the consensus that the remarkable performance of microdroplets correlates to their unique interface properties (68, 69).

### Photo-induced inorganic carbon transformation progress analysis

**Fig. 4.**
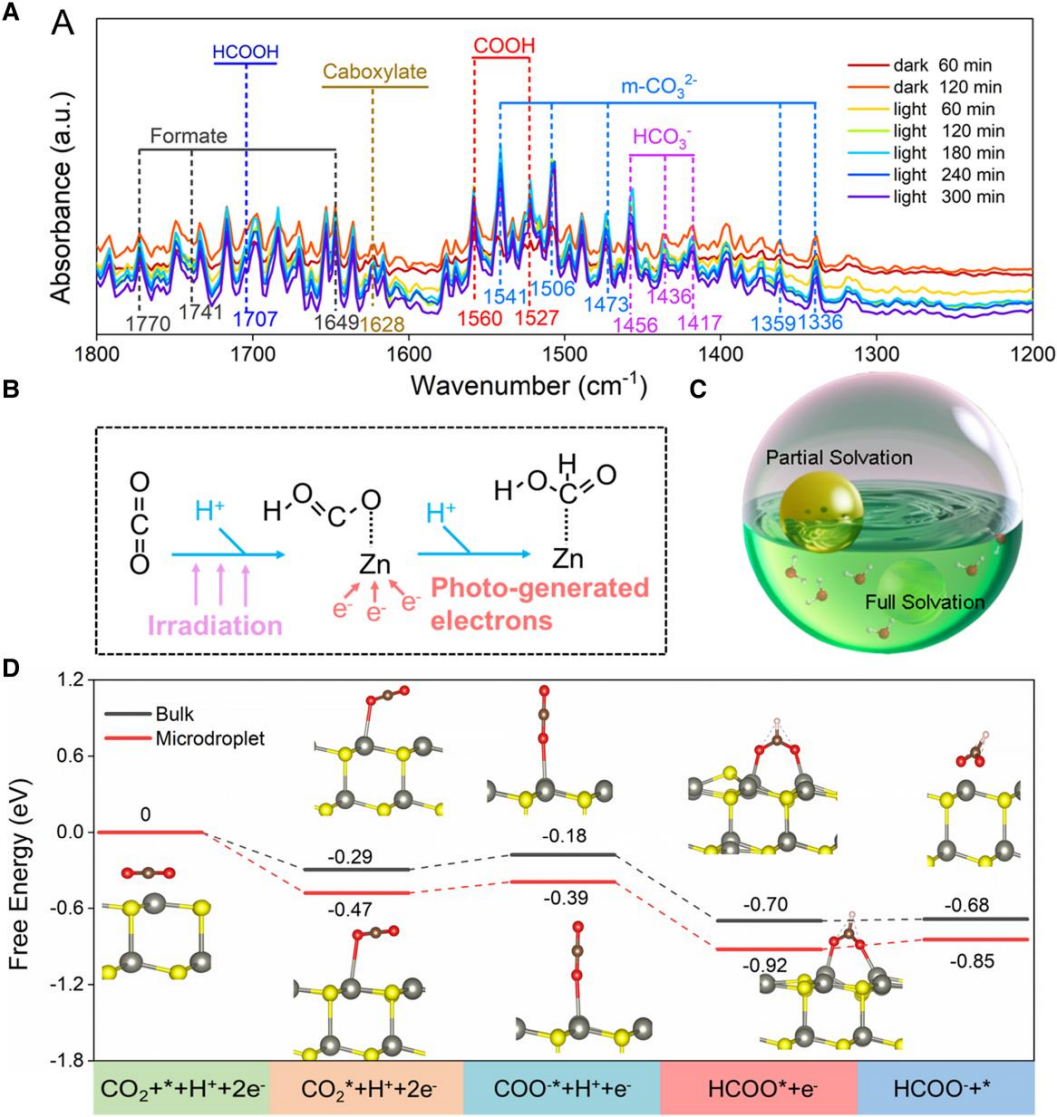
ZnS-mediated inorganic carbon reduction pathways. A) In situ DIFTS spectra for the photoreduction reaction. Diagrams of B) reaction pathway and C) partial solvation effect. D) Free energy diagram for the pathway of CO_2_ conversion into HCOOH in microdroplet system and bulk phase reaction, indicating the electron/proton transfer process at the (001) surface of ZnS.

Before we set out to specify the complicated ZnS-bearing microdroplet reaction system, the basic mechanism of photo-induced inorganic carbon transformation on the surface of ZnS was first explored using in situ Diffuse Reflaxions Infrared Fourier Transformations Spectroscopy (DRIFTS) analysis (Figs. 4A and S11) (70–75). There are several bands featured at 1,541, 1,506, 1,473, 1,351, and 1,330 cm^−1^, corresponding to the characteristic vibration of C=O[ν(C=O)] and monodentate carbonate (m-CO_3_^2−^) (76), respectively. Meanwhile, these bands centered at 1,456, 1,436, and 1,417 cm^−1^ are indicative of the presence of adsorbed bicarbonate (HCO_3_^−^), which is reported to be an intermediate species that is responsible for the subsequent HCOOH formation over the ZnS surface (77). Accumulating uptake of CO_2_ and H_2_O molecules on the ZnS surface leads to a significant increase in the abundance of (bi)carbonate species. The increase observed in the bands at 1,649, 1,741, 1,761, and 1,770 cm^−1^ verify the presence of HCOOH, and the band at 1,628 cm^−1^ is indicative of carboxylate formation. The intensity of the bands observed at 1,560 and 1,527 cm^−1^, corresponding to *COOH, shows a steady growth as illumination time prolongs. This trend can be potentially attributed to the strong proton capture capability of CO_2_^•−^ radicals. Of utmost significance, the *HCOOH band at 1,707 cm^−1^ gradually increases as the light-induced reaction proceeds. These species are crucial intermediates in HCOOH formation. Specifically, the combination of band energy structure calculations with in situ DRIFTS analysis, we can deduce that upon solar irradiation the photogenerated electrons and holes are separated and transferred to the Zn and S of natural ZnS mineral, followed by the subsequent occurrence of redox reaction. On the Zn site, the conversion of CO_2_ to HCOOH involves two consecutive hydrogenation steps, with the first step involving the conversion of CO_2_ into *COOH, followed by a secondary reaction in which the formed *COOH undergoes secondary transformation to eventually yield HCOOH. On the other hand, the oxidation of H_2_O at the S site involves multiple conversion steps, when HCOOH is desorbed from the system, the two OH^•^ radicals quench themselves to generate O_2_ and H_2_O, thus maintaining the charge balance of the catalytic system. In Fig. 4A and B, the formation of *COOH and OH^•^ from CO_2_ and H_2_O is the rate-determining step in the process of the photocatalytic overall reaction (77, 78). In addition, two intermediates, *COOH and HCOO*, are produced in the first hydrogenation reaction. The *COOH is thermodynamically favorable since the energy of the formation of *COOH is lower than that of HCOO*. It is noteworthy that the energy required for the dehydration of *COOH into CO is considerably higher than that of *COOH hydrogenation into HCOOH. This observation is in line with the experimental observations, which demonstrate the HCOOH of high selectivity among the reduction products. Overall, the reaction pathway comprises three elementary steps. Initially, the chemisorbed CO_2_ or HCO_3_^−^ accumulates on the ZnS surface. In the subsequent step, the occurrence of electron transfer upon excitation is accompanied by the HCOO* generation and contributes to the formation of HCOO^−^ through a second electron transfer event. Finally, HCOO^−^ desorbs from the catalyst surface to complete a typical redox cycle. As the semisolvation effect is the fundamental driving force to trigger fast solar-driven CO_2_ reduction in the “microdroplet” reaction chamber of our concern (detailed in Discussion section), reaction processes and intermediates involved are routinely considered to be the same (32, 34, 79), although CO_2_ reduction to HCOOH occurs in air–water interface and bulk medium, respectively.

### Thermodynamic aspect concerning HCOOH production in microdroplets suspended with sphalerite

Recall that the unique dynamic behavior of the solvation shell at the interface can alter the charge transfer efficiency or the redox capability (68), which has been evidenced to play a crucial role in organic synthesis in a “microdroplet” reaction chamber. On this basis, one can expect that photochemical redox will be strengthened for sphalerite resided in the microdroplet interface. Generally, a large solvent energy barrier must be overcome in the bulk solution of multiple solvation shells, which greatly slows down the reaction (80). In stark contrast, for interfacial solute molecules of the incomplete solvent cage, i.e. semisolvation, the heterolysis of the covalent bond will contribute to the fast separation of charges of electron–hole pairs emitted from sphalerite upon irradiation, which can further reduce the energy of the transition state of CO_2_RR intermediates, leaving the activation energy of the reaction being reduced and the reaction speed being greatly accelerated (81). In light of this, DFT in conjunction with the projector-augmented wave method is applied to calculate the change in Gibbs free energy (Δ*G*) both in bulk and microdroplet reaction and further to gain a deep insight into the reaction mechanism behind the significant yield of HCOOH in a ZnS-bearing microdroplet system of pronounced partial solvation effects (the details are available in Materials and methods section) (32, 33, 58, 82).

Figure 4C and D shows the thermodynamic energetics of the key reaction intermediates involved in the microdroplet and bulk ZnS suspension systems. CO_2_ accepts one electron given by the ZnS mineral to produce COO^−*^ and the free energy increases, suggesting that the first electron transfer from the ZnS surface to CO_2_ is the rate-determining step. Clearly, when the reaction occurs at the air–water interface, the partially solvated reactant and TS of more localized charges reduce the difference between the energy of reactant (CO_2_*) and TS (COO^−^*) from 0.11 to 0.08 eV and thus increase the reaction rate constant at the interface accordingly. The promising “microdroplet” reaction system observed for HCOOH production relative to the bulk phase reaction is consistent with our early laboratory-based observation (9). When the photoreduction reaction occurs in the microdroplet, the activation barrier of the overall reaction process can be significantly reduced and thus beneficial for HCOOH production. The Δ*G* of CO_2_ reduction to HCOOH in bulk solution is −0.68 eV, while it is −0.85 eV in microdroplets, which means that microdroplets alter the kinetics and thermodynamics of photoreduction inorganic carbon. In bulk solution, large solvation-energy barriers that are required for reactions to occur should be overcome, which greatly slows the reaction rate, while at the air–water interface, reduced desolvation energy is required for the reagent with an open solvent shell. The semisolvation effect gives rise to the molecular organization and alignment of reactants at the air–water interface of the microdroplet surface, thus contributing to the reduction of the entropic change (33). This result agrees well with the early finding where the phosphorylation of ribose is thermodynamically unfavorable (5.6 kcal mol^−1^), while it shows Gibbs free energy of −1.1 kcal mol^−1^ in the microdroplet reaction system of the same reaction process (32). The underlying mechanism can be explained by the enlarged bond dipole and ordered orientation of reactant molecules residing at air/water interface, which therefore reduce the entropy of the initial state and increase the free energy of the initial state (68). A similar observation of favorable energetic variation has been found for the nonnegligible affinity of free OH radicals toward the air–water interface, which instantaneously alters electrophilicity/nucleophilicity, HOMO (Highest Occupied Molecular Orbital)–LUMO (Lower Unoccupied Molecular Orbital) gap, and other reactivity indicators of the complex to trigger the fast reaction within the incomplete solvation shell (83–85). Overall, we observe that the microdroplet chamber had a reduced entropic cost compared with the reaction in the bulk aqueous system. This explains the extremely high yield of formic acid during the photochemical process, where the ZnS mineral in the droplet efficiently converts inorganic carbon to HCOOH.

### Implications for microdroplet photoreactive mineral in initial prebiotic Earth systems

**Fig. 5.**
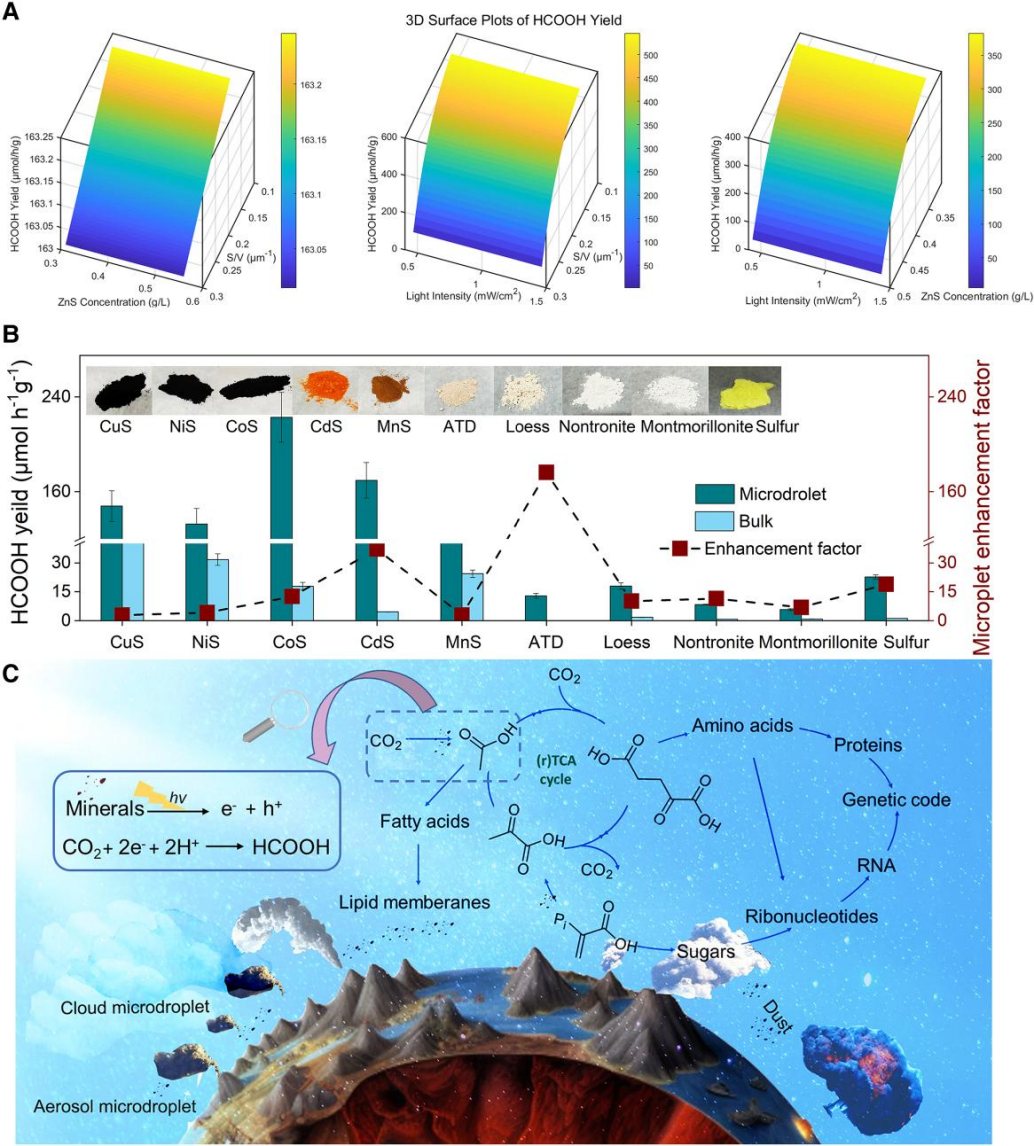
Cumulative yields of HCOOH in the proposed model. A) The cumulative HCOOH depends on *I* (light intensity), *S_V_* (microdroplet *S*/*V*), and *C*_ZnS_ (concentration of ZnS). (50 mM carbonate ions, pH = 7, and 1.48 mW cm^−2^ UV wavelength was 310 nm.) B) Photoreduction CO_2_ on various minerals within microdroplet. C) Schematic diagram of abiotic carbon fixation in the primitive Earth.

In the early prebiotic Earth systems, the total flux of intense ultraviolet C (UVC) radiation is eight times greater than that at the present age, with abundant aerosol water estimated to be approximately 1,000-fold that of the contemporary Earth’s atmosphere (86). Mineral dust can enter into aerosol or cloud microdroplets and their absorption of extreme UVC has laid the foundation for the occurrence of life in the primitive Earth (Fig. 5C). To estimate the abiotic production of organic molecules and evaluate the role of the ZnS microdroplet proposed in this work, an ideal numerical calculation by supposing that ZnS particles suspended in the aerosol microdroplet was performed. The functional relationships between the HCOOH yield (*Y*_HCOOH_) and ZnS concentration (*C*_ZnS_), surface-to-volume of microdroplet (*S*/*V*), and light intensity (*I*) were derived regression analysis of condition dependence in laboratory-based measurement (Eqs. 6–8, Figs. 3F inset and S12–S14). These parameters altogether determine the total yield of HCOOH in mineral ZnS droplets during the atmospheric photochemical process. Based on the above results, we simulated the yield of formic acid under the joint coupling influence of three factors, namely different mineral concentrations, different *S*/*V* or microdroplet sizes, and different light intensities (Fig. 5A, calculation details are available in the Materials and methods section). Notably, in early Earth-relevant conditions, the amount of HCOOH produced by the numerical calculation is dependent on ZnS concentration (*C*_ZnS_), surface-to-volume microdroplet (*S*/*V*), and light intensity (*I*). The production of HCOOH from inorganic carbon in this pathway is higher comparable with other organics with higher molecular weight like formaldehyde, hydrogen cyanide, and amino acids, which have been documented as other abiotic conversion pathways of inorganic carbon, e.g. impact shocks, and lightning, respectively. Accordingly, based on the determined correlations between yield and *I*, *S*/*V*, and *C*_ZnS_ (Eqs. 9–11), we estimated an extremely high HCOOH yield up to 2.518 × 10^14^ kg year^−1^ by applying the parameters reported for early Earth conditions (1). More significantly, the conversion of HCOOH to other complicated biomolecules has a reducing potential of −0.11 V (vs. NHE at pH 7) for methane and 0.07 V for acetic acid. The multistep synthesis of macromolecules based on HCOOH is thermodynamically favorable in the natural universal “microdroplet” chambers comprising mineral sphalerite.

We further explored the acceleration effect for microdroplet and bulk reaction systems regarding the inorganic carbon photoreduction process across a wide range of authentic minerals and clays (Fig. 5B). Clearly, all minerals and clays, i.e. CuS, NiS, CoS, CdS, MnS, elemental sulfur, Arizona Test Dust (ATD), loess, nontronite, and montmorillonite, show remarkable enhancement when they exist in the microdroplet system relative to the bulk one, typically 1–2 orders of magnitude. This observation further emphasizes the generality of the naturally synthesized process by inorganic carbon source for biomolecular origin within mineral-bearing microdroplet reaction systems, which may be even more significant than we are now investigating here and thus provide an alternative and efficient abiogenic pathway to produce biomolecules considering the significant increase in HCOOH yield in the microdroplets relative to the conventional bulk phase reaction regardless of the types of authentic minerals and clays (Fig. 5B). In the early Earth's atmosphere, the formic acid produced within the aerosol or cloud microdroplets can undergo subsequent photochemical processes to generate fatty acids. These fatty acids, in turn, have the capability to form vesicle structures, creating a relatively closed space that facilitates the formation of more complex life-building reactions. On the other hand, diverse metrics of aerosol particles full of natural minerals may potentially convert atmospheric CO_2_ to esters, analogous to the acetyl-Coenzyme A pathway. These esters can further be involved in the synthesis of essential biomolecules, such as amino acids and ribonucleotides, thus offering the ideal building blocks for the emergence of life (Fig. 5C).

## Discussion

In summary, the natural prebiotic reactions utilizing inorganic carbon in the aerosol microdroplet of abundant air–water interface contribute to an unrecognized high yield of HCOOH, which is over two orders of magnitude higher than that in bulk phase reaction. This can be explained by the decrease in entropy change of chemical reactions in microdroplets of semisolvation effect, where the molecular structure and arrangement of reactants at the air–water interface allow the reaction more thermodynamically favorable compared with the bulk phase. Notably, on the contemporary and early Earth, aqueous microdroplets and airwater interfaces are ubiquitous in the natural environments, including the surface of oceans, lakes, and hydrothermal vents. As an important part of the reaction of the origin of life in hydrothermal vents, mineral aerosol microdroplets extensively exist in hydrothermal vents (87) and play an important role in the process of the origin of life reaction. Moreover, the integrated surface area of aqueous aerosol particles that partially come from the ocean can be several orders of magnitude larger than the surface area of the ocean itself. In the primitive Earth environment, more than 90% of the Earth’s surface was covered by oceans (8), and the interface abundance of aqueous atmospheric aerosol particles was more than that found in nowadays conditions. During their lifetime, atmospheric aerosols experience a complicated aging process, with variations in temperature, relative humidity, pH, salinity, solar light exposure as well as interaction with other gas-phase species, and undergo diverse physical processes including coagulation, hydration, and dehydration. In this case, large quantities of water–air interfaces over oceans, lakes, aerosols, and cloud and fog droplets essentially provide favorable microenvironments for the natural prebiotic synthesis. During the photochemical processes of the origin of life in the natural airborne mineral-bearing droplet particles, the activation energy barrier of the overall reaction process can be significantly reduced in the microdroplet system, thus remarkably promoting the production of basic biomolecule block HCOOH than what previously considered. Additionally, this work potentially provides a new perspective on the element cycle of sulfur and carbon. Specifically, airborne mineral dust-bearing microdroplet particles serve as a highly efficient “natural” carbon and sulfur storage system in the primitive Earth environment. On the one hand, atmospheric carbon dioxide from geologic activity can be efficiently transformed into the basic organic molecule within the aerosol particles, and these carbon sources will eventually be brought back to the ocean and continental land through the dry or wet deposition process. At the same time, a minor fraction of the “naturally synthesized” multicarbon organic compounds within these airborne microdroplets is speculated to undergo a photolysis process in response to ultraviolet light radiation, resulting in the formation of inorganic carbon that subsequently becomes an integrated part of the atmospheric carbon cycle. On the other hand, photochemically, sulfide minerals are documented to be excellent hole sacrificial agents during the CO_2_RR process, thus leading to the formation of sulfur oxides or by-product sulfate. This sulfur mineralization process contributes to atmospheric sulfur dioxide and leads to the sulfur cycle in primitive atmospheres. To conclude, the microdroplet photochemical reaction regarding the origin of the life system provides a novel perspective and significant abiogenic pathway to understand biomolecule production in the early Earth environment.

## Materials and methods

### Preparation of ZnS sample

Natural mineral samples were ground into powders by a ball-grinding mill. Colloidal ZnS suspensions were prepared based on a previous method. The dropwise addition of 100 mL of 50 mM Na_2_S·9H_2_O (99.1% assay; Aladdin) to 100 mL of 50 mM ZnSO_4_·7H_2_O took place under stirring and purging with N_2_ (g). Ultrapure water purged with N_2_ was used in every step. The ZnS colloidal suspension was centrifuged at 4,400 rpm for 5 min (Eppendorf 5702) and rinsed twice with water to remove excess sulfate prior to irradiation.

### Preparation of other metal sulfides

All metal sulfides were prepared by dropwise addition of 100 mM Na_2_S into a 100-mM aqueous solution of the corresponding metal chloride (CdCl_2_, CoCl_2_, CuCl_2_, MnCl_2_, NiCl_2_) under vigorous stirring with a final volume ratio of 1:1. Solid precipitates were then separated from the supernatant solution by centrifugation and dried under vacuum. To prevent oxidation by atmospheric O_2_, the sample preparation was conducted in a glove box filled with N_2_ gas. All chemicals were purchased from Aladdin as reagent grade and were used without further purification.

### Characterization of sphalerite

XRD patterns were recorded using a Bruker D8 Advance diffractometer at Shiyanjia Lab (www.shiyanjia.com). The HAADF-STEM images and the corresponding EDS mapping of catalysts were performed on an FEI Tecnai F20, Japan, at Shiyanjia Lab (www.shiyanjia.com). The light absorption properties of the powder samples were recorded on a UV–vis spectrometer (SHIMADZU UV-2600). Mott−Schottky test was investigated by a three-electrode system, Na_2_SO_4_ (1 M) solution as the electrolyte, Ag/AgCl electrode as the reference electrode, the platinum mesh as the counter electrode, and conductive glass with the sample to be measured as the working electrode. In a certain voltage range (1 V), the test frequency was changed (generally 500 and 1,000 Hz).

### In situ DRIFTS

To probe the inorganic carbon catalytic transformation over the mineral sphalerite, the in situ DRIFTS (IRTracer-100; Shimadzu Instrument Corporation) analysis was conducted. Those DRIFTS spectra ranging from 4,500 to 900 cm^−1^ were collected by using a high-sensitivity mercury cadmium telluride detector with a resolution of 4 cm^−1^ for 100 scans and recorded by diffuse reflectance accessory. Thermocouple wires attached to the ceramic sample holder and a temperature controller were used, allowing resistive heating from a UV lamp (wavelength = 310 nm) during the reaction. Moreover, a cooling circulation device was applied considering the temperature fluctuation of samples due to the light source. All measurements were carried out at 298 K. Prior to the reaction gas entering the cell, further pretreatment was performed by sending dry airflow to the DRIFTS chamber under the dark for 1 h to remove the contamination residual on particle samples. The DFIRT spectra were obtained by subtracting the sphalerite background.

### Evaluation of photovoltaic efficiency

The IPCE in the photochemical experiment under monochromatic light was estimated according to the yield of HCOOH, using Eq. 1


(1) $$\mathrm{IPCE}=\frac{n_{e}}{n_{p}}=\frac{2\;\times \;N_{\mathrm{HCOOH}}\;\times \;N_{A}}{\frac{P_{\mathrm{light}}\;\times \;t\;\times \;A}{hv}}\;\;\;\;\;\;\;\;\;\;\;\;\;\;\;\;\;\;\;\;\;\;\;\;\;\;\;$$

where *n_e_*, *n_p_*, *N*_HCOOH_, *N_A_*, *P*_light_, *t*, *A*, *h*, and *v* are the number of electrons for HCOOH production from inorganic carbon, the number of incident photons, the mole number of produced HCOOH, Avogadro constant, the power of monochromatic light reaching reactor, irradiation time, the exposed area of the reactor, Planck constant, and the frequency of monochromatic light, respectively.

### Band gap calculations

*E_g_* of samples were obtained from the UV–vis spectra with a Tauc plot:


(2) $$\left(\alpha hv{)}^{\frac{1}{2}}=A(hv-E_{g}\right)$$

Here, *α* is the absorption coefficient, *h* is the Planck constant, *v* is the frequency, *A* is the constant, and *E_g_* is the band gap. Data processing was further done by Kubelka–Munk theory and Einstein photochemical equivalence law: *F*(*R*) = (1 − *R*):


(3) $$F(R)=\frac{{\left(1\;-\;R\right)}^{2}}{2R}$$

(4) hv=hc/λ

Among these parameters, *R* is the absorbance, which can be obtained from the UV–Vis DRS. *λ* is the wavelength of light, and *c* is the speed of light. The curve is obtained by taking *hv* (eV) on the *x*-axis and *F*(*R*) on the *y*-axis. *E* is obtained by the *x*-axis value of the intersection of the tangent of the *F*(*R*)−*E* curve and the line with *y* = 0.

### Photochemical experiments on inorganic carbon reduction

All experiments were conducted under simplified conditions of a microdroplet ZnS reaction system, wherein synthesized colloidal ZnS, the concentration of sodium bicarbonate (NaHCO_3_), levels of CO_2_ concentration, and light intensity were all taken into consideration. Unless otherwise specified, the uniform condition was set as ZnS (0.8 g L^−1^), 25 mM NaHCO_3_. The experimental and blank groups (without ZnS, light, or carbon source) were performed in triplicate. First, the microdroplet was produced by the sprayer and microdroplet printer (jetlab II, MicroFab) on an as-prepared superhydrophobic quartz wafer (the contact angle was about 142°, Fig. 2). The solar-driven reaction chamber (schematic diagram available in Fig. S6) enables inorganic carbon source gas-phase CO_2_ or aqueous bicarbonate to be properly introduced into the reaction system before light experiments. In Fig. S3, the photocatalysts were still uniformly dispersed in the microdroplets after standing for 180 min, which confirmed the great dispersal of catalysts during the photocatalysis reaction and the feasibility of microdroplet photocatalysis. (Note: a black tetrafluoroethylene hydrophobic carbon paper was used for microdroplet observation.) For bulk phase photocatalysis experiments, 3 mL of colloidal solution was added into a quartz Petri dish (diameter = 30 mm). The photoreduction of inorganic carbon in microdroplets was performed using a custom-designed quartz reactor that can maintain high humidity (90%) and low temperature (Fig. S6). At a given time interval, the microdroplets were then collected and quantitatively diluted using deionized water for further analysis. The products in the liquid phase were analyzed by IC (using a Metrohm 883 Basic IC system equipped with a Metrosep A supply 5-250/4.0 analytical column and a conductivity detector), and the gaseous products were detected using a GC-TCD instrument (Shimadzu GC-2014) with a 5-Å molecular sieve packing column. Other metal-sulfide minerals, as well as loess, ATD, montmorillonite, and nontronite, were dispersed into water (concentration of 1 g L^−1^), and then produced by sprayer droplets in the abovementioned reactor (25 mM NaHCO_3_, light intensity 1.48 mW cm^−2^, high humidity 90%, 310 nm).

### Raman measurement

The Raman measurements were performed with an XploRA Plus confocal Raman spectrometer (Jobin Yvon, HORIBA, Gr, France) coupled with a ×10 Olympus microscope objective (Olympus, 0.9 Numerical Aperture). The size-dependent HCOOH yield using the Raman analysis, where a confocal Raman spectroscopy coupled with an optical microscope (RENISHAWS inVia Raman microscope). External-cavity diode laser (532 and 785 nm) was used for excitation. The Raman signal was collected using a multichannel electron-multiplying charge coupled device ranging from 400 to 4,200 cm^−1^, with two spectrum accumulations at a 40-s acquisition time per spectrum. The Raman data analyses including baseline removal by a polynomial equation and spectra fitting with the Gauss–Lorenz function were conducted using LabSpec 6 software. Then the inorganic carbon reduction reaction process was monitored by the Confocal Raman spectrometer in real-time.

### ^13^C isotopic substitution experiment

^13^CO_2_ and ^12^CO_2_ bubbled into ZnS colloidal suspension (0.8 mg L^−1^) for 30 min. The obtained ^13^CO_2_-saturated and ^13^CO_2_-saturated solutions are the stock solutions to produce microdroplets for photocatalytic reaction in the designed reactor (Fig. S9). Before the light irradiation, the reactor was purged for 10 min by ^13^CO_2_ and ^12^CO_2_, respectively. The microdroplet was detected by the confocal Raman spectrometer after a reaction of 180 min under 310 nm UV-lamp radiation.

### DFT calculation details

First-principle calculations were carried out based on periodic DFT using a generalized gradient approximation within the Perdew–Burke–Ernzerh of exchange correction functional. The DFT calculation considers unsolved reactants which already reported that the calculated value is very close to the real value for the reaction in aqueous microdroplets (33) and fully solvated reactants (bulk reaction). The wave functions were constructed from the expansion of plane waves with an energy cutoff of 450 eV. Gamma-centered *k*-point of 2 × 2 × 1 has been used for geometry optimization. The consistency tolerances for the geometry optimization are set as 1.0 × 10^−6^ eV atom^−1^ for total energy and 0.05 eV Å^−1^ for force, respectively. In order to avoid the interaction between the two surfaces, a large vacuum gap of 15 Å has been selected in the periodically repeated slabs. In free energy calculations, the entropic corrections and zero-point energy (ZPE) have been included.

The free energy of species was calculated according to the standard formula:


(5) ΔG=E+ΔZPE+ΔH−ΔTS

where Δ*H* is the integrated heat capacity, *T* is the temperature of the product, and *S* is the entropy.

### Numerical calculation description and the estimation of organic production

The relationship between HCOOH concentration and *C*_ZnS_: ZnS emitted into the atmosphere at a rate of up to 5.638 Tg year^−1^. Assuming that the concentration of ZnS minerals in cloud water and aerosols ranges from 0.21 to 0.55 mg L^−1^ (81).


(6) $$Y=441.59+9.64\;\times \;e^{\left(\frac{\left(C_{\mathrm{ZnS}}-10\right)}{16.28}\right)}+9.64\times 2\times e^{\left(\frac{C_{\mathrm{ZnS}}-10}{19.90}\right)}$$

The relationship between HCOOH yield (*Y*, μmol h^−1^ g^−1^) and *S*/*V* (*S_V_*, μm^−1^) is given as


(7) $$Y=2250.61+((622\;-2250.61))/\left(1+{\left(\frac{S_{V}}{241.43}\right)}^{7.39}\right)$$

The relationship between HCOOH yield (*Y*, μmol h^−1^ g^−1^) and light intensity (*I*, mW cm^−2^) is written as


(8) $$Y=27.43\;\times \;e^{\left(\frac{I}{0.39}\right)}$$

The relationship between HCOOH yield (*Y*, μmol h^−1^ g^−1^), light intensity (*I*, mW cm^−2^), and *S_V_* (μm^−1^) is written as


(9) $$\begin{array}{rl} \left(Y,C_{\mathrm{ZnS}},S_{V}\right) & =1809.02\;-\;9.64\;\times \;e^{\frac{C_{\mathrm{ZnS}}-10}{16.28}}+2\;\times \;e^{\frac{C_{\mathrm{ZnS}}-10}{19.90}}\\ & +\frac{622-2250.61}{1+\;{\left(\frac{S_{V}}{241.43}\right)}^{7.39}} \end{array}$$

(10) $$(Y,S_{V},\;I)=2223.18-27.43\;\times \;e^{\frac{I}{0.39}}+\frac{622-2250.61}{1+{\left(\frac{S_{V}}{241.43}\right)}^{7.39}}$$

(11) $$(Y,C_{\mathrm{ZnS}},I)=414.16+9.64\;\times \;e^{\frac{C_{\mathrm{ZnS}}-10}{16.28}}+2\;\times \;e^{\frac{C_{\mathrm{ZnS}}-10}{19.90}}+27.43\;\times \;e^{\frac{I}{0.39}}$$

All relevant inputs employed for the numerical model are summarized in Table S1.

## Supplementary Material

pgad389_Supplementary_Data

## Data Availability

All data are available in the manuscript and Supplementary material.
