# Supplementary material for: Prebiotic synthesis of mineral-bearing microdroplet from inorganic carbon photoreduction at air–water interface
Source: PNAS Nexus. 2023 Nov 15;2(11):pgad389. doi: 10.1093/pnasnexus/pgad389 (PMC10682977; doi:10.1093/pnasnexus/pgad389)
Supplement: pgad389_Supplementary_Data [file pgad389_supplementary_data.docx]

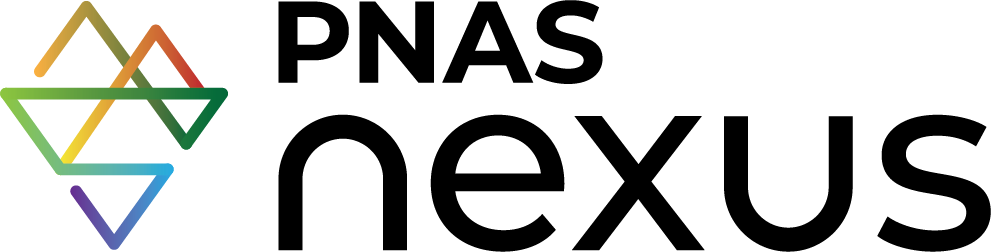


**Supporting Information for**

Prebiotic synthesis from inorganic carbon photoreduction at air-water interface of mineral-bearing microdroplet

Qiuyue Ge^a^, Yangyang Liu^a,^*, Wenbo You^a^, Wei Wang^a^, Kejian Li^a^, Xuejun Ruan^a^, Lifang Xie^a^, Tao Wang^a^, Liwu Zhang^a,b,^*

^a^Shanghai Key Laboratory of Atmospheric Particle Pollution and Prevention, National Observations and Research Station for Wetland Ecosystems of the Yangtze Estuary, IRDR International Center of Excellence on Risk Interconnectivity and Governance on Weather, Department of Environmental Science & Engineering, Fudan University, Shanghai, 200433, P. R. China.

^b^Shanghai Institute of Pollution Control and Ecological Security, Shanghai, 200092, P. R. China.

*To whom correspondence may be addressed. **Email:** [yangyangliu@fudan.edu.cn](mailto:yangyangliu@fudan.edu.cn), [zhanglw@fudan.edu.cn](mailto:zhanglw@fudan.edu.cn)

**This PDF file includes:**

Figures S1 to S14

Table S1


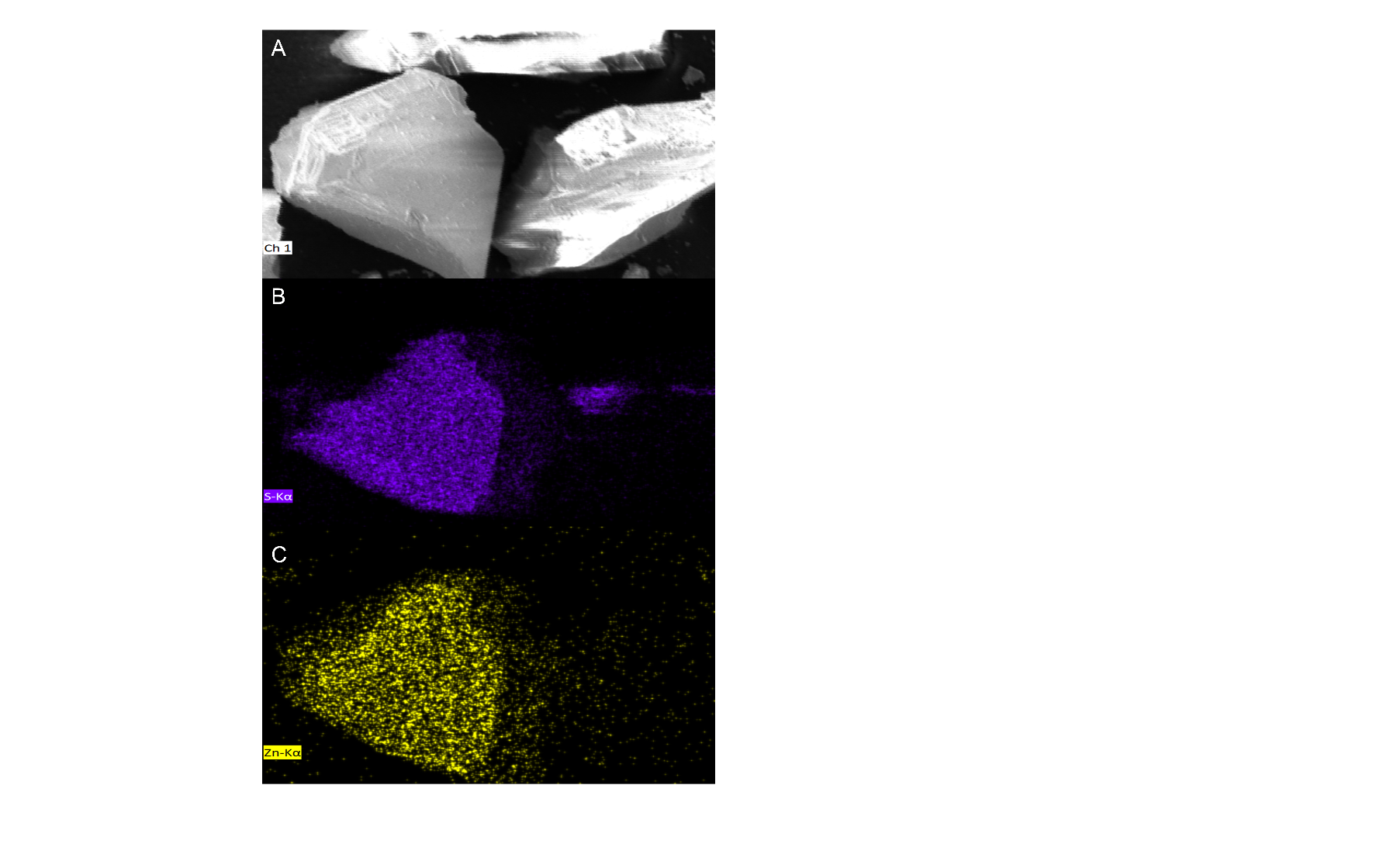


Figure S1. Scanning electron microscopy (SEM) of natural sphalerite mineral. (A) SEM image and EDS mapping of element (B) S, (C) Zn.





Figure S2. Characterization of synthesized ZnS. (A) XRD pattern of ZnS.(B)The corresponding bright-field TEM, HAADF-STEM, and EDS analysis of the compositional elements (HRTEM images, the lattice fringe is 0.317 nm corresponding to (111) crystal plane).


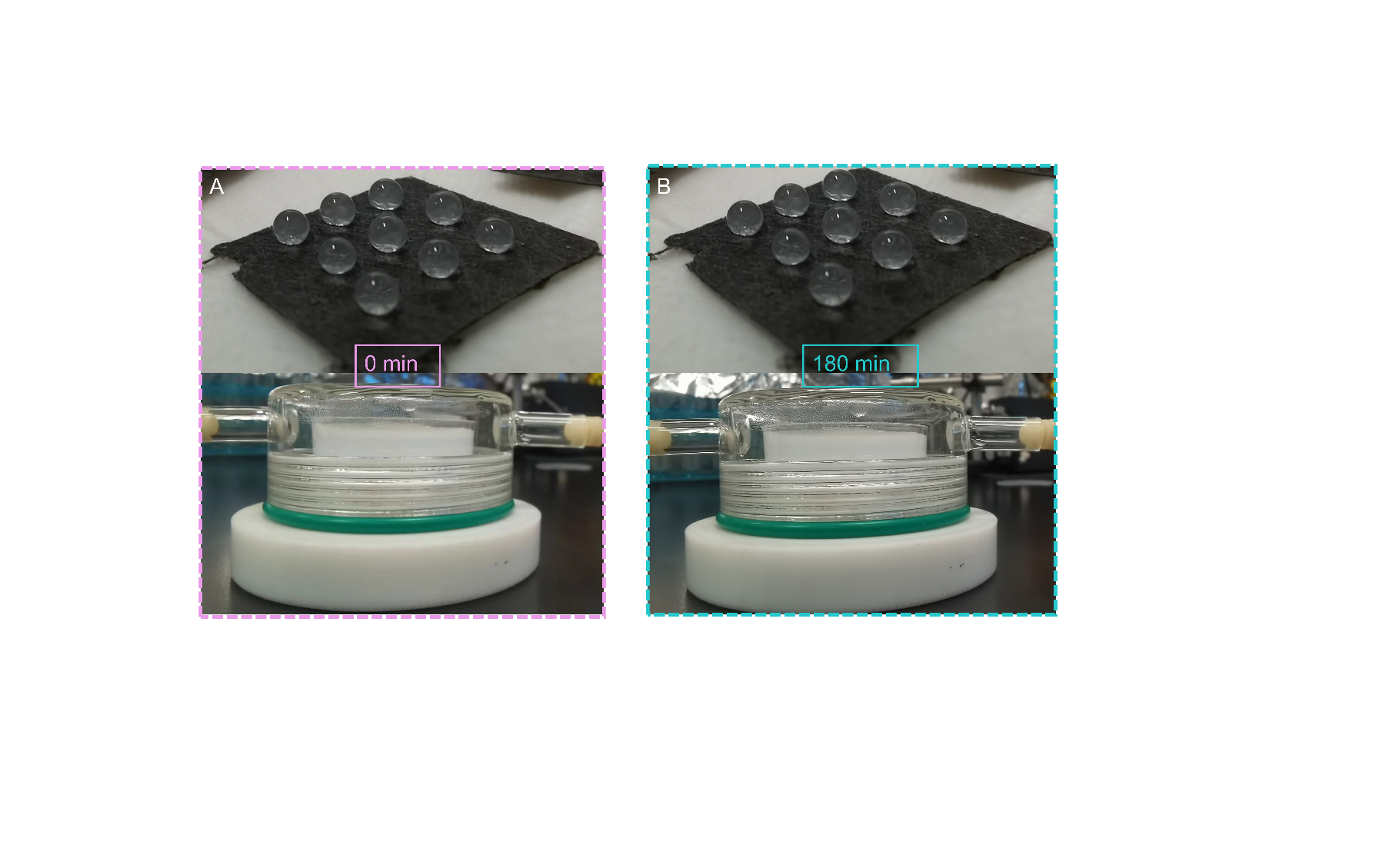


Figure S3. Images of microdroplets being stabilized at different time scales. The microdroplets were used to investigate the stability of the catalyst homogeneous dispersion in water microdroplets before (A) and after (B) 180 min.

**
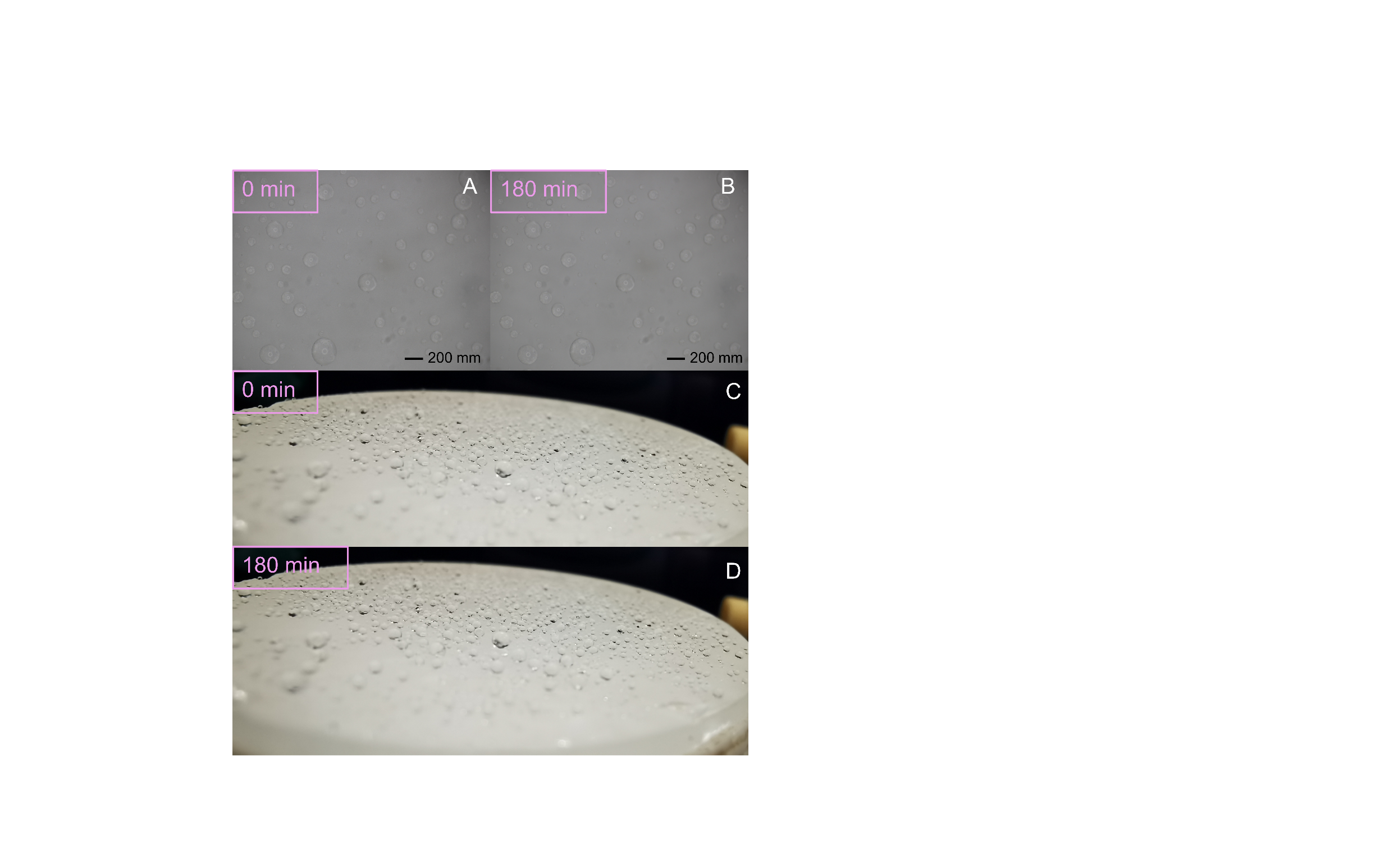
**

Figure S4. The photo of microdroplets with varying diameters produced by the sprayer. The photomicrographs and camera photos of the microdroplet before (A, C) and after (B, D) 180 min. The insets are the corresponding water contact angle of superhydrophobic high-humidity gas flow (RH = ~90%) was obtained by passing the fresh gas through a bottle containing 80% vol. deionized water.

**

**

Figure S5. Potochemistry reduction inorganic carbon underdifferent condition.


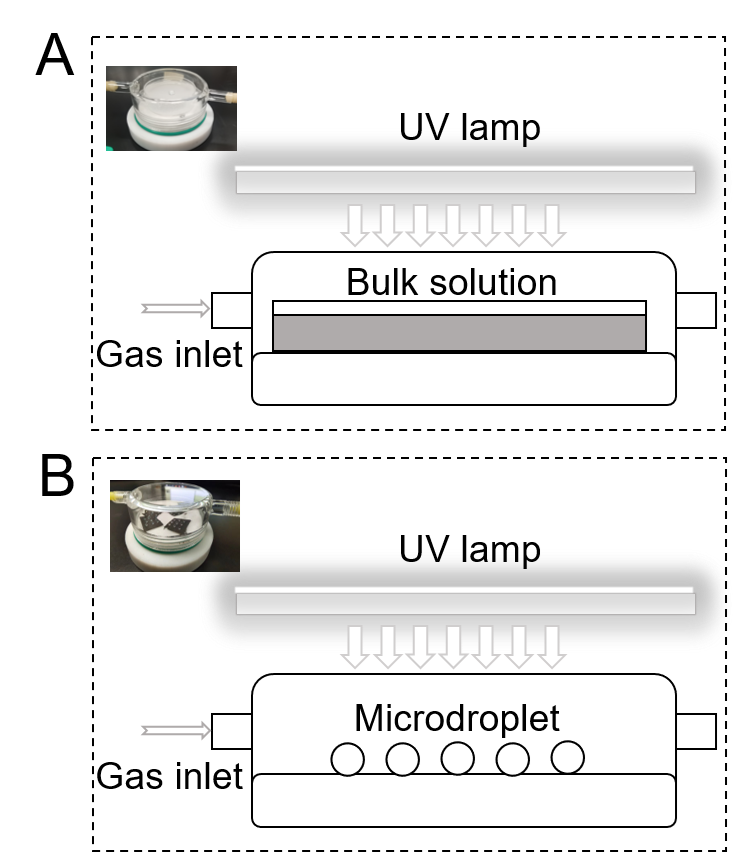


Figure S6. The schematic diagram of photoreduction inorganic carbon reaction system. (A) Bulk phase reaction in the designed quartz reactor (Inset: the photo of the bulk phase reaction in the designed quartz reactor). (B) microdroplet reaction (Inset: the photo of the microdroplet reaction in the designed quartz reactor).





Figure S7. Bulk phase photoreduction inorganic carbon pre-experiment. Bulk phase reaction under different inorganic carbon sources. (310 nm UV lamp radiation, room temperature, 180 min, 0.8 g L^-1^ ZnS, 25 mM NaHCO_3_).


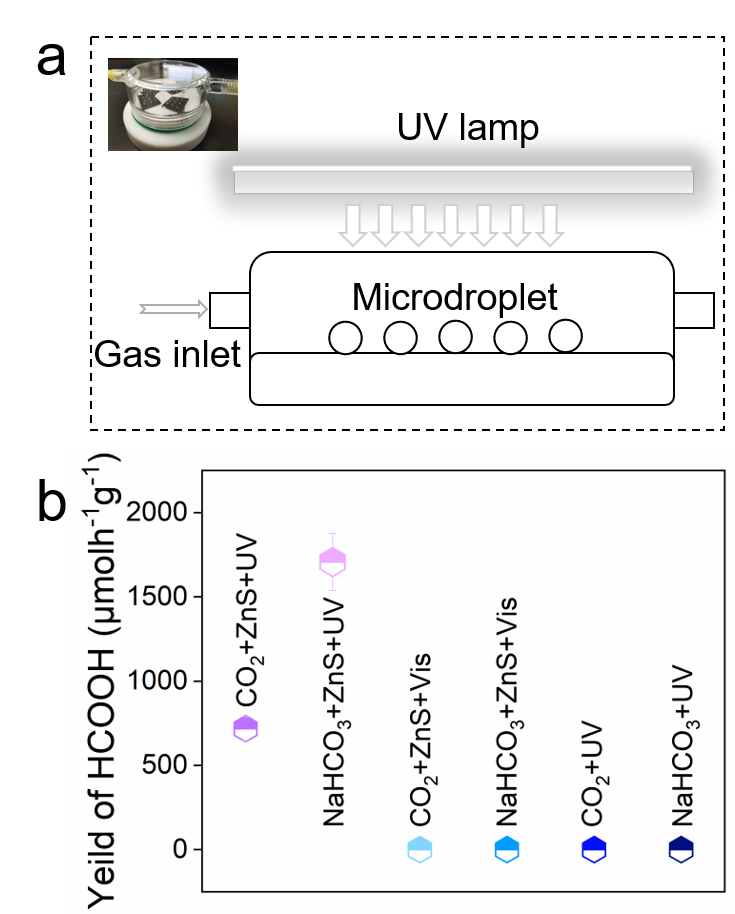


Figure S8. Control experiment of microdroplet system. Control experiment of microdroplet system under different conditions which were the absence of the ZnS, UV light radiation, and inorganic carbon source.





Figure S9. ^13^CO_2_ isotope experiment in microdroplet. Raman spectra analysis of HCOOH that was derived from the photocatalytic reduction of ^13^CO_2_ (^12^CO_2_ when necessary as a reference).


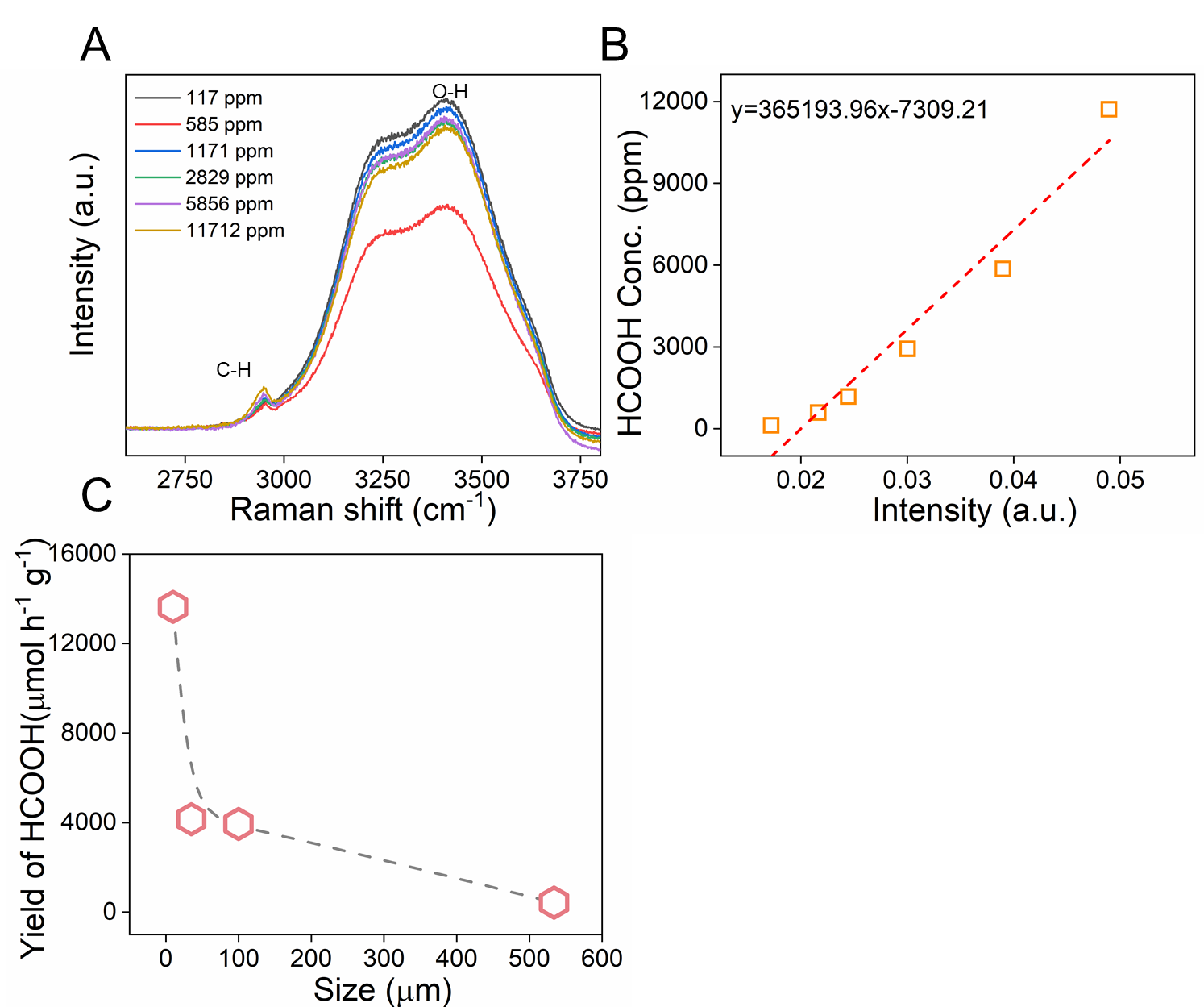


Figure S10. Quantification of HCOOH yield by applying the Raman spectrometer. (A) Raman spectra of different HCOOH concentrations and (B) the calibration curves built upon the HCOOH concentration and Raman intensity of the characteristic peak. (C) The size-dependent yield of HCOOH within the microdroplet reaction system (diameter range: 10-534 μm).

**

**

Figure S11. The schematic diagram of in-situ FTIR experiments





Figure S12. Time-dependence photocatalytic HCOOH production in NaHCO_3_-system. The initial NaHCO_3_ concentration is 25 mM both in bulk phase and microdroplet reaction. (UV lamp radiation, room temperature, 0.8 g L^-1^ ZnS).





Figure S13. ZnS concentration-dependence photocatalytic HCOOH production in NaHCO_3_-system. The initial NaHCO_3_ concentration is 25 mM both in bulk phase and microdroplet reaction. (UV lamp radiation, room temperature,).





Figure S14. Light intensity-dependence photocatalytic HCOOH production in NaHCO_3_-system. The initial NaHCO_3_ concentration is 25 mM both in bulk phase and microdroplet reaction. (UV lamp radiation, room temperature,).

Table S1. Water content in the atmosphere.

| Title | Value (g/cm^3^) | Ref |
| --- | --- | --- |
| Troposphere aerosol water | 3.5955 × 10^14^ | (1) |
| Cloud water | 1.79775 × 10^19^ | (2, 3) |
| Total water | 1.79779 × 10^19^ | (2, 3) |

**SI Reference**

1. Lin G*, et al.* 2014. Global modeling of SOA: the use of different mechanisms for aqueous-phase formation. *Atmos Chem Phys.* 14(11): 5451-5475.

2. Zhang RY*, et al.* 2015. Formation of Urban Fine Particulate Matter. *Chem Rev.* 115(10): 3803-3855.

3. Zhang K*, et al.* 2012. The global aerosol-climate model ECHAM-HAM, version 2: sensitivity to improvements in process representations. *Atmos Chem Phys.* 12(19): 8911-8949.
